# Neuroendocrine and Metabolic Effects of Low-Calorie and Non-Calorie Sweeteners

**DOI:** 10.3389/fendo.2020.00444

**Published:** 2020-07-16

**Authors:** Eleonora Moriconi, Alessandra Feraco, Vincenzo Marzolla, Marco Infante, Mauro Lombardo, Andrea Fabbri, Massimiliano Caprio

**Affiliations:** ^1^Laboratory of Cardiovascular Endocrinology, IRCCS San Raffaele Pisana, Rome, Italy; ^2^Section of Medical Pathophysiology, Food Science and Endocrinology, Department of Experimental Medicine, Sapienza University of Rome, Rome, Italy; ^3^Unit of Endocrinology and Metabolic Diseases, Department of Systems Medicine, CTO A. Alesini Hospital, University of Rome Tor Vergata, Rome, Italy; ^4^Department of Human Sciences and Promotion of the Quality of Life, San Raffaele Roma Open University, Rome, Italy

**Keywords:** body weight, microbiota, safety, obesity, diabetes, sugar, metabolic health, VLCKD

## Abstract

Since excessive sugar consumption has been related to the development of chronic metabolic diseases prevalent in the western world, the use of sweeteners has gradually increased worldwide over the last few years. Although low- and non-calorie sweeteners may represent a valuable tool to reduce calorie intake and prevent weight gain, studies investigating the safety and efficacy of these compounds in the short- and long-term period are scarce and controversial. Therefore, future studies will need to elucidate the potential beneficial and/or detrimental effects of different types of sweeteners on metabolic health (energy balance, appetite, body weight, cardiometabolic risk factors) in healthy subjects and patients with diabetes, obesity and metabolic syndrome. In this regard, the impact of different sweeteners on central nervous system, gut hormones and gut microbiota is important, given the strong implications that changes in such systems may have for human health. The aim of this narrative review is to summarize the current evidence for the neuroendocrine and metabolic effects of sweeteners, as well as their impact on gut microbiota. Finally, we briefly discuss the advantages of the use of sweeteners in the context of very-low calorie ketogenic diets.

## Introduction

On the basis of their energy content, sweeteners can be classified into calorie, low-calorie and non-calorie compounds. Calorie-sweeteners include natural sugars (1), such as sucrose, glucose, fructose, maltose, lactose, and trehalose. They are mainly present in fruits, honey, milk, dairy products, and mushrooms (2) and their caloric values is on average 4 kcal/g. Their sweetening power is measured in relation to sucrose, which is considered as a reference sugar (3). Low calorie and non-calorie sweeteners provide no or few calories and are characterized by a high sweetness taste. Low-calorie sweeteners include polyols or sugar alcohols, which are low-digestible compounds obtained from the replacement of an aldehyde group with a hydroxyl one (4). The most common polyols are sorbitol, xylitol, maltitol, mannitol, erythritol, isomalt, and lactitol; they are naturally found in fruits, vegetables, and mushrooms (5). Non-calorie sweeteners are mostly obtained by chemical synthesis (except Stevia rebaudiana), and are characterized by minimal or absent nutritional content (3). They include saccharin, aspartame, acesulfame-k, and sucralose (6).

In the last few decades, intake of sugar (free and added sugars) is dramatically increased, especially in western world (7). High intake of sugars has been related to the development of several diseases, including obesity, type 2 diabetes, cardiovascular disease, non-alcoholic fatty liver disease (8–10), as well as tooth decay (11), neurocognitive diseases (12), and chronic inflammatory disorders (13).

In 2015, the World Health Organization (WHO) recommended the consumption of free sugars below 10% of total daily energy intake. However, a further reduction in free sugars intake below 5% of total energy intake has been strongly suggested (14). For a healthy adult, 5% of total energy intake is equivalent to ~25 g of sugar per day. Free sugars include all sugars added to foods by the manufacturer, as well as sugars naturally present in non-intact fruit and vegetables (i.e., juiced or pureed). Free sugars do not include sugars naturally present in intact fruit, vegetables, and dairy products (15).

With regard to children and adolescents, a scientific statement published by the American Heart Association (AHA) in 2017 recommends <25 g of added sugars per day, although added sugar should not be included in the diet for children <2 years of age (16). According to a recent study, in UK added or free sugar intake has been estimated between 7% and 13% of total energy intake, respectively, and it is higher in children than in adults (17). The prevalence of obesity and its comorbidities (such as type 2 diabetes, cardiovascular diseases and cancer) has dramatically increased (18) and several governments started to promote policies aimed to encourage a healthy diet and lifestyle (19). In 2011 Denmark introduced a tax on saturated fat, which was repealed in 2012, since it demonstrated a positive but not consistent effect on health (20). In the same year, Hungary added levy on foods with high fat, sugar, salt and caffeine content; soft drinks and alcohols were also taxed. In 2012, France introduced a tax on sweetened beverages (21, 22). In United States, where sweetened beverages consumption is still high, taxes on sugar-sweetened beverages have been approved since 2014 (23, 24). In California, the first state which approved the tax, a 21% reduction of sweetened beverage consumption was reported (25) and a positive impact on health care cost savings was observed (26). Since 2016, UK approved several policies in order to reduce childhood obesity, including the “sugar tax” on sweetened beverages, called the Soft Drinks Industry Levy, which became effective in April 2018 (27). In Hungary, sugar tax resulted in a qualitative improvement and reformulation of food products (28, 29).

In addition, nutrition labeling has been encouraged in order to help consumers to choose healthy foods. In 2013, the Food Standards Agency in the UK promoted a color coding in labeling system. In this color coding system, red, yellow, and green labels correspond to high, medium, and low percentages of fat, salt, sugar, and total energy present in the product, respectively (30). In this regard, some studies found that front-of-package nutrition labels can readily convey to consumers key information on the nutritional profile of different food products, showing that green labels are associated with the highest healthfulness perception of these products (31). However, Vasiljevic et al. found that nutritional labels of snack foods had limited impact on perceptions of healthiness and no effects on the snack choice, whereas emoticon labels had stronger effects on perceptions of taste and healthfulness of snacks compared to color labels (32).

A recent Cochrane Review evaluated the effects exerted in the general population by the taxation of unprocessed sugar or sugar added foods in terms of consumption of these foods and changes in prevalence and incidence of overweight, obesity, and other diet-related diseases (33). The authors concluded that there is still limited (and low-quality) evidence to support that taxing unprocessed sugar or sugar added foods has a significant impact on reducing their consumption and preventing overweight, obesity or other adverse health outcomes (33). Therefore, future studies are needed to draw concrete conclusions in this direction. Notably, an article by Fernandez and Raine (34) recently reviewed the impact of sugar-sweetened beverage taxation on obesity, concluding that current evidence is still limited. Importantly, authors suggest that sugar-sweetened beverage taxation will likely fail to have a significant impact on the prevalence of obesity and associated non-communicable diseases until this policy will not be associated with interventions aimed to increase access to non-sweetened beverages, educate consumers about healthy beverage alternatives and explore taxation of other beverages and non-nutritive foods (34).

To address the growing health issue of obesity, sweetener consumption has gradually increased over the last years (35–38). Indeed, when used judiciously, non-calorie sweeteners may facilitate reductions in the intake of added sugars, leading to decreased total energy, weight loss, prevention of weight gain and/or subsequent beneficial effects on related metabolic parameters (38). Nonetheless, these potential benefits may not be fully achieved without reductions in total food intake and/or in presence of a compensatory increase in energy intake from other sources (38). In addition, animal and human studies have reported controversial results on the safety of non-calorie sweeteners (39). Besides their potential to reduce daily calorie content, non-calorie sweeteners were reported to potentially display detrimental metabolic (weight gain) (40) and neuroendocrine (addiction) effects (41). Conversely, some intervention studies reported that consumption of non-calorie sweeteners is associated with weight loss and improved metabolic parameters (42, 43). Despite a growing use of non-calorie sweeteners, which is gradually increasing in both healthy and obese/overweight individuals, there is indeed a knowledge gap regarding their safety and efficacy in the long term-period. Therefore, there is an urgent need to update the current positions from international agencies on the use of these compounds.

We will review here the metabolic and neuroendocrine properties of the most commonly used low-calorie (polyols) and non-calorie sweeteners, along with their safety profile and main use in food industry.

## Low-Calorie Sweeteners (Polyols or Sugar Alcohols)

Sugar alcohols (also referred to as polyols) are characterized by a lower calorie content (2 to 4 kcal/g) (44) than sucrose (Table 1) (45). Low amounts of polyols are naturally present in vegetables, mushrooms and fruits (melon, peach, apple, pear, apricot), but also in oat (50, 51). They include hydrogenated mono-, di-, oligo-, and polysaccharides (45) and are mainly used in “sugar-free” products, sweets, and chewing gums (52). Polyols are stable compounds at high temperatures and do not interfere in the Maillard reaction (53). The late stages of Maillard reaction lead to the generation of the so-called AGEs (also referred to as “advanced glycation end-products”) in foods and biological systems (54, 55). Of note, AGEs contribute to the development of micro- and macrovascular complications of diabetes (56–58), by inducing oxidative stress and activating inflammatory pathways (59, 60) Polyols do not affect glucose homeostasis (61, 62). Also, polyols have long been suggested as valid sugar substitutes able to exert a beneficial role on insulin resistance and glucose control in patients with type 2 diabetes and/or metabolic syndrome. Nonetheless, robust evidence on the long-term effects of polyols in terms of glucose control and chronic complications in diabetic patients is still scarce and inconclusive (45). Interestingly, most of these compounds do not undergo fermentation by oral bacteria flora (63); therefore, polyols can reduce the risk of tooth decay because they represent a poor source of energy to resident bacteria of the oral cavity and do not create an acidic environment (45, 64).

**Table 1:** Comparative profile of the main calorie sweeteners and low-calorie sweeteners^a^.

**Sweetener**	**Glycemic index^b^**	**Caloric value (kcal/g)^c^**	**Sweetening power^d^**	**EFSA code^e^**
**SUGARS**				
Glucose	100	4	0.75	
Fructose	23	4	1.7	–
Sucrose	65	4	1	
Lactose	45	4	0.15	
Maltose	105	4	0.3	
**POLYOLS (SUGAR ALCOHOLS)**				
Erythritol	0	0.2	0.6–0.8	E-968
Sorbitol	9	2.7	0.5–0.7	E-420
Mannitol	0	1.6	0.5–0.7	E-421
Xylitol	13	2.4	1	E-967
**CALORIE NATURAL SWEETENERS**				
Trehalose	45–50	3.6	0.5–0.7	–
Thaumatin	0	4	2,000–2,500	E-957

a *Source (45, 46)*.

*EFSA, European Food Safety Authority*.

b *Glycemic index (GI) represents the blood glucose response measured as area under the curve (AUC) in response to a test food consumed by an individual under standard conditions, expressed as a percentage of the AUC after consumption of a reference food (usually 50 g glucose) consumed by the same individual on a different day. According to the most commonly used GI classification, foods are categorized as having a low ( ≤ 55), medium (55–69), or high GI (≥70) (47)*.

c *Source (1, 48)*.

d *Source (45)*.

e *Source (49)*.

Polyols increase saccharolytic anaerobic and aciduric bacteria in the colon and give rise to the production of short-chain fatty acids which play a key role in the maintenance of the intestinal epithelial barrier (45). Although acceptable daily intake (ADI) dose has not been established for polyol increased polyol consumption may cause gastrointestinal discomfort and laxative effects in healthy individuals (61, 64). The European Union legislation approved the use of seven different polyols, including erythritol, isomalt, lactitol, maltitol, mannitol, sorbitol, and xylitol (49).

Herein, we summarize the main properties of the polyols that are most commonly used in food and beverage industry, also discussing their potential impact on human health.

### Sorbitol (E-420)

Sorbitol provides 2.6 kcal/g. Sorbitol is naturally present in grapes, prunes, cherries, peaches, apples, pears, and fruit juices (65). Sorbitol is poorly absorbed in the small intestine, while in the colon it is converted by gut microbiota into gases and short-chain fatty acids, providing energy (66). Sorbitol has osmotic effects and it acts as a laxative when ingested in high doses (20–50 g) (67). In addition, chronic ingestion of sorbitol through chewing gums can cause increased intestinal motility regardless of its osmotic effect (68). Therefore, sorbitol use should be avoided by individuals with irritable bowel syndrome (68). In 1993 FDA approved sorbitol use as Generally Recognized As Safe (GRAS) (69).

### Mannitol (E-421)

Mannitol is naturally present in mushrooms, marine algae, strawberries, onions, and pumpkins (70). Only 25% of ingested mannitol is absorbed in the gut, whereas the remaining part is excreted in the urine. In the gut, mannitol is slowly fermented (45). Mannitol is virtually inert and does not interfere with pharmacological compounds. Due to this reason, it is used also in hygiene products, drug filler and intravenous fluid solutions (53). Moreover, the osmotic diuretic properties of mannitol account for its use as intravenous solution in the management of elevated intracranial pressure and cerebral edema (71).

### Xylitol (E-967)

Xylitol is a natural sweetener found in fruits, vegetables and oats, and it is extracted from birch trees (5). Due to its low caloric content (2.5 kcal/g) and low glycemic index, xylitol has long been suggested as a valid alternative to glucose and sucrose in patients with diabetes (72). In a pre-clinical study, 4 week administration of xylitol at high doses has been shown to improve glucose tolerance in rats (73). Conversely, a randomized, placebo-controlled, crossover trial conducted in lean and obese volunteers showed that acute xylitol and erythritol ingestion did not significantly affect circulating levels of glucose and insulin, despite being able to stimulate the secretion of the gut hormones cholecystokinin (CCK) and glucagon-like peptide-1 (GLP-1). Of note, the marked increase in CCK and GLP-1 levels was accompanied by a significant slowing in gastric emptying (74). These findings are interesting and do not exclude that chronic ingestion of these sweeteners may play a role in the regulation of glucose homeostasis. Also, the increase in GLP-1 levels may have relevant clinical implications beyond the insulinotropic action of GLP-1, considering the well-known role exerted by GLP-1 receptor agonists in the reduction of cardiovascular risk among diabetic patients, along with the anorexigenic properties of GLP-1 and its analogs (75–77). Remarkably, CCK has also been shown to induce short-term satiety and to play a role in the regulation of insulin secretion and overall β-cell function and survival, displaying complementary biological actions with those exerted by GLP-1 (78).

### Erythritol (E-968)

Erythritol is a polyol contained in fruits (e.g., melon, peach), wine and beer (79). It is chemically derived from the fermentation of natural sugars (e.g., glucose and sucrose) by Trichosporonoides megachiliensis (80). Its sweetening power corresponds to 60–80% of that of sucrose (81). Erythritol is poorly absorbed in the jejunum and is excreted unmodified in the urine (82). Only a small fraction of erythritol undergoes gut fermentation. Therefore, an excessive consumption of erythritol can be associated with laxative effects (83). Gastrointestinal discomfort is generally observed when erythritol intake is >1,000 mg/kg of body weight (79). Erythritol intake does not appear to have detrimental effects on glucose control and its use is generally deemed as safe in patients with diabetes (84).

Similarly to other polyols, erythritol does not participate in Maillard-type reactions and, therefore, does not cause the production of AGEs. In addition, by acting as a scavenger for hydroxyl radicals, erythritol exerts anti-oxidant and endothelium-protective properties (83). Erythritol provides a negligible amount of energy (0.2 kcal/g) (64). Thus, it is commonly used as part of the dietary patterns recommended for people with obesity (85). Due to its sweet taste and high digestive tolerance, and the fact that it is virtually calorie-free and non-cariogenic, erythritol is widely used in the food and beverage industry.

## Non-Calorie Sweeteners

Non-calorie sweeteners (also known as artificial sweeteners or non-nutritive sweeteners) are defined as compounds with high sweetening power. Although most of them do not provide calories upon ingestion, some of these compounds (such as aspartame and stevia rebaudiana) have a measurable caloric value that is considered negligible at the doses commonly used by humans.

Non-calorie sweeteners can be of synthetic or natural origin (Table 2).

**Table 2:** Comparative profile of the main non-calorie sweeteners approved by the European Food Safety Authority.

**Sweetener**	**Brand Names^a^**	**ADI (mg/kg body weight/day)^b^**	**Sweetening power^c^**	**EFSA code^d^**
Acesulfame-K	Sweet One Sunett	15	200	E950
Aspartame	Nutrasweet Equal	40	200	E951
Saccharin	Sweet and Low Sweet Twin Necta Sweet	5	300-500	E954
Sucralose	Splenda	5	600	E955
Steviol glycosides	Truvia	4	200-300	E960

*ADI, Acceptable daily intake*.

*EFSA, European Food Safety Authority*.

a *Source (86, 87)*.

b *Source (86, 87)*.

c *Source (86, 87)*.

d *Source (88)*.

### Stevia Rebaudiana (E-960)

Stevia rebaudiana has a natural origin. It is commonly called Stevia and derives from a plant that grows in South America (89). Stevia contains steviol glycosides, stevioside, and rebaudioside A, that account for its sweet taste, and other minor glycosides, such as rebaudioside B, rebaudioside C, rebaudioside D, rebaudioside E, rebaudioside F, dulcoside A, rubusoside, and steviolbioside. Stevia also contains a complex of terpenes, tannins, sterols, vitamins, carotenes, flavonoids, and other microelements (90). After ingestion, the steviol glycosides contained in Stevia are not digested in the upper gastrointestinal tract (91), but they are metabolized by bacteria of the Bacteroidaceae family in the colon, resulting in the production of steviol (92), which is subsequently processed in the liver and converted into steviol glucuronide (93). Energy from fermentation of steviol glycosides (usually assessed as 2 kcal/g) is low (92).

Stevia has a strong sweetening power, 200- to 400-fold higher than that of sucrose (94). Its maximal recommended daily intake is 4 mg/kg, and it is considered unsafe at higher doses (EU regulation 1129/2011) (95). This quantity corresponds to approximately nine tablets per day. Considering that stevia is 200 to 400 times sweeter than sugar, it is extremely unlikely for an individual to ingest the maximum dose of 4 mg/kg over a 24 h period. Stevia offers several advantages over other non-calorie sucrose substitutes. *In vitro*, stevia displayed anti-viral effects (96), immunomodulatory activity (97) and anti-inflammatory properties, through inhibition of NF-κB and pro-inflammatory cytokines expression (98). In rats, stevioside showed antihyperglycemic effects through the enhancement of the first-phase of insulin secretion with a concomitant suppression of glucagon levels; stevia also caused a pronounced reduction of both systolic and diastolic blood pressure in rats (99).

Intriguingly, pre-clinical evidence suggests that steviol glycoside derivatives can exert antiproliferative properties in several cancer cell lines, including pancreatic (100), breast (101), and gastric cancer (102) cell lines.

### Aspartame (E-951)

Aspartame was discovered in 1965 (103). It provides 4 kcal/g, but it is included in the group of non-calorie sweeteners, due to its strong sweetening power (104). It is composed by phenylalanine, aspartic acid and methanol (105). Given the high content in phenylalanine, aspartame use is contraindicated in individuals with phenylketonuria, a rare autosomal recessive inborn error of metabolism characterized by a decreased metabolism of the amino acid phenylalanine (106).

Although the use of aspartame has been approved in United States since 1974 (107) and in Europe since 1994 (108), its safety is still debated. After several studies performed during the 1970s and the 1980s (109–112), a long term study was carried out in rats to assess its carcinogenic potential (113). Rodents treated with different dosages of aspartame until their natural death showed an increase in the frequencies of lymphomas and leukemias, carcinomas of the renal pelvis and ureter, and schwannomas (114). These results were confirmed even at doses of 20 mg/kg body weight, which are lower than the recommended maximum daily intake in Europe and in United States (115). The potential carcinogenicity of aspartame was first attributed to methanol, that is converted into formaldehyde and then into formic acid both in rats and humans (116). Based on data obtained from different studies, the European Food Safety Authority (EFSA) was called in 2013 to re-evaluate the safety of aspartame on human health. EFSA concluded that aspartame is safe at a dose of 40 mg/kg body weight/day (117). However, aspartame safety on human health is still under debate; in fact, a recent study highlighted several important shortcomings in the EFSA document (118).

### Acesulfame-K (E-950)

Acesulfame-K is a potassium salt of 6-methyl-123-axanthiazine-4 (3H)-one 2,2 –dioxide. Its sweetening power is 120-fold higher than sucrose (119). Acesulfame-K does not provide calories. Since it is not catabolized in humans, acesulfame-K does not affect serum potassium levels despite its potassium content (50). The acceptable daily intake (ADI) of acesulfame-K is 15 mg/kg body weight. It is used in various sweet foods and beverages (119).

Hydrolysis of acesulfame-K gives rise to acetoacetamide, a degradation product that can be toxic if produced in large amounts (120). Acesulfame-K carcinogenicity has been investigated in rats, where no carcinogenic effects were observed (121). The majority of studies noted that it displays neutral effects on body weight or glucose tolerance (122, 123).

### Sucralose (E-955)

Sucralose is derived from sucrose after replacement of three chloride atoms with three hydroxyl groups (124). It was discovered in 1976 and has a sweetening power 450- to 650-fold higher than sucrose. The ADI of sucralose is 5 mg/kg body weight in United States (125) and 15 mg/kg body weight in Europe (126). Only up to 11–27% of sucralose is absorbed in the gastrointestinal tract, while the remaining undergoes intestinal excretion unmodified (127). Sucralose is stable during baking and it is considered safe in beverages and foods that require cooking (128). Sucralose consumption does not affect glycemic control or insulin sensitivity in healthy individuals when administered alone, whereas its use in combination with carbohydrates showed a negative impact on glucose metabolism (129).

Several studies *in vitro* (130, 131) and *in vivo* (132, 133) demonstrated that sucralose is not a carcinogenic compound. Only two studies noted a positive relationship between sucralose and mutagenic activity. In one, two human colon cancer cell lines (Caco-2 and HT-29) and one human embryonic kidney cell line (HEK-293) were exposed to very high doses of sweetener solutions for up to 24, 48, and 72 h, leading to cell alterations and DNA fragmentation (134). In the other, mouse lymphoma cells showed doubtful results when exposed to 10 mg/ml sucralose concentrations (131). However, in both studies very high sucralose concentrations were used. Recently, Soffritti et al. raised questions regarding sucralose safety (135), although EFSA revaluation judged that these results were not supported by the available data (136).

### Saccharin (E-954)

Saccharin is a non-calorie sweetener derived from 1,2-benzoisothiazol 3-(2H). Its sweetening potency is almost 300-fold higher than that of sucrose. Saccharin has an unpleasant bitter or metallic taste (106). Experiments conducted in the 1980s have showed a link between saccharin and an increased incidence of bladder cancer in a rat strain genetically susceptible to bladder tumors, when exposed to 5% saccharin in the diet for 52 weeks (137). Nonetheless, saccharin generates a urinary precipitate mainly composed of calcium phosphate, which can exert cytotoxic effects on urothelial cells of rats and induce mild hyperplasia (138). However, very high saccharin concentrations were tested in animal models, if compared to the doses commonly ingested by humans (139). With regard to metabolic parameters, a recent study evaluated the administration of saccharin (at different doses, namely: 2.5, 5, and 10 mg/kg) in male Wistar rats (140). An increased body weight was noted in rats after 60 and 120 days of 5 mg/kg saccharin treatment. Authors also observed an increase in glucose, uric acid and creatinine levels, as well as in oxidative status in the liver of saccharin-treated rats, suggesting that saccharin may impair glucose homeostasis, induce obesity and lead to impairments in kidney and liver function (140). The World Health Organization and the EU Scientific Committee for Food declared saccharine as safe up to the approved daily intake doses (5 mg/kg body weight) (53). Nowdays, saccharine is commonly used in soft drinks, baked foods, jams, canned fruit, candy, dessert toppings, and chewing gum (141).

## Impact of Sweeteners on Central Nervous System and Metabolic Outcomes

The consequences of low-calorie and non-calorie sweeteners on daily food consumption and eating behavior are still controversial. Eating causes an amplification of dopamine release in the nucleus accumbens, similar to what occurs upon substance abuse (142). However, the ingestion of palatable foods causes an increase in dopamine production greater than standard food (143). Given that palatable foods stimulate the same neural pathways involved in drug addiction, it has been suggested that an excessive sugar intake can lead to addiction. Moreover, after long-term consumption of sugar, withdrawal symptoms have been described in rats, similarly to what has been observed in morphine and nicotine dependence (144). Food addiction leads to changes in the expression of dopamine receptors (145). Neuroimaging studies revealed that obese individuals exhibit lower dopamine sensitivity in nucleus accumbens accompanied by a decrease in dopamine D2 receptor expression, similar to what has been observed in drug-addicted subjects (146–149). Interestingly, dopamine D2 receptor expression is inversely related to body mass index in obese patients (150). These findings suggest that chronic exposure to sugar decreases dopamine-D2 receptor expression. Furthermore, it has been hypothesized that obese subjects respond to dopamine deficiency by overeating palatable foods (151).

Several brain regions are involved in food-reward, namely: lateral hypothalamic area (LHA), ventral tegmental area (VTA), nucleus accumbens (NAc) and prefrontal cortex (PFC). Several neurotransmitters (GABA, glutamate and opioids) are involved in different aspects of reward in the above-mentioned brain regions (152, 153). In particular, the dopaminergic circuitry from LHA to VTA and from VTA to the NAc is involved in hedonic processes (“liking”), reinforcement (“learning”), and motivation (“wanting”) (154), while acetylcholine is involved in the aversive aspects of withdrawal (155). During withdrawal state, extracellular dopamine decreases in the accumbens, while acetylcholine is released from accumbens neurons. Intermittent or excessive sugar consumption induces neurochemical modifications, mimicking the effects of opioids (144). Food choice and food intake are physiologically regulated by metabolic and neural signals. In particular, metabolic signals act as nutritional status sensors and mediate the ingestion of a sufficient amount of energy. On the other hand, sensory signals regulate food choice and are linked to subsequent metabolic adaptation, resulting in conditioned responses to these foods (156). The combination of learned responses with metabolic and sensory signals results in a specific pattern of food intake. The responses to sensory inputs, such as taste, texture, and sight of food, include consecutive preabsorptive physiological responses, which are collectively referred to as cephalic phase responses. Such digestive preparation confers to the body the ability to anticipate the particular challenge a food poses for maintaining energy homeostasis (157). Among these responses, the cephalic-phase insulin response, elicited by sugars, enhances glucose tolerance in humans (158–160).

Although replacing calorie with non-calorie sweeteners definitely reduces the energy density of foods and beverages, this does not necessarily translate into metabolic advantages and improved health status. It has been hypothesized that daily intake of non-calorie sweeteners can “trick” the brain by encouraging sugar craving and addiction (161). Indeed, lack of calories generally abolishes the post-ingestive food reward mediated by the hypothalamus (162). In keeping with this, it has been suggested that uncoupling sweet taste from energy causes progressive weakening of conditioned responses to sweet taste (163). As previously mentioned, sweet taste is able to evoke physiological adaptations which play an important role in the finely-tuned regulation of energy homeostasis, by sensing the presence of caloric nutrients in the gut and facilitating the absorption and subsequent utilization of energy. When sweeteners are not associated with caloric intake, their ability to sense energy is altered, with a subsequent reduced ability to use energy and a mitigated activation of the peripheral and central pathways that promote the feeling of satiety (163).

**Figure 1:**
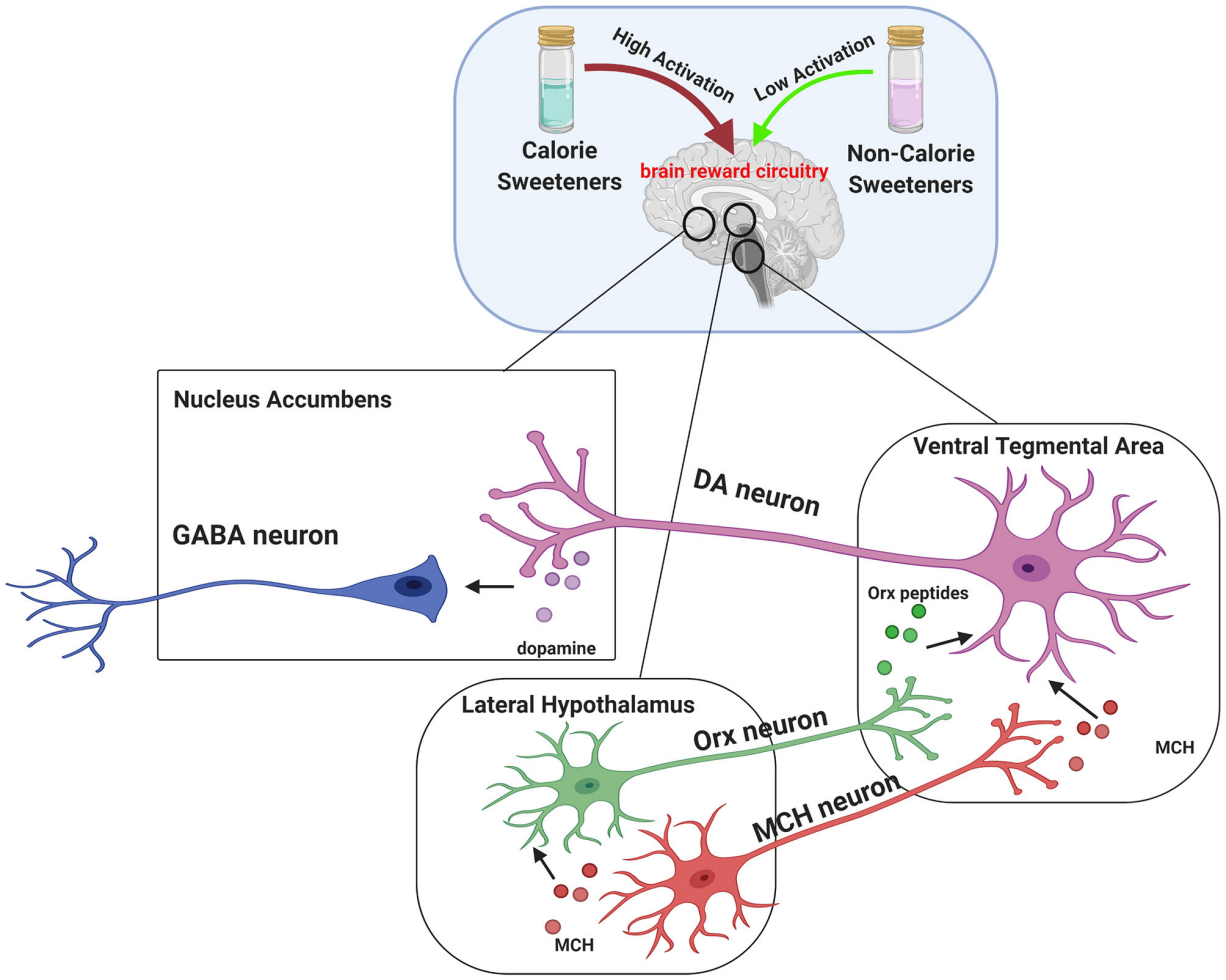
Brain reward circuitry involved in central effects of sweeteners. The dopaminergic pathway is strictly involved in hedonic processes (“liking”), reinforcement (“learning”), and motivation (“wanting”). Midbrain dopaminergic circuits include Lateral Hypothalamus (LHA), Ventral Tegmental Area (VTA), and Nucleus Accumbens (NAc). Dopamine release is driven by orexin (ORX) peptides and melanin-concentrating hormone (MCH) secreted by LHA. In particular, ORX and MCH neurons from LHA project to VTA, where Orx peptides and MCH mediate the activation of dopamine (DA) neurons and increase the release of DA in projection areas such as the NAc. It has been established that dopamine reward pathway response induced by caloric sweeteners consumption, such as sucrose, is greater compared to non-calorie sweetener sucralose. Interestingly, a preclinical study provided evidence that MCH neurons account for the natural preference for sucrose over sucralose and that such effect can be reversed by stimulating MCH neurons with light. This suggests that non calorie-sweeteners require additional stimuli to obtain the same rewarding effect of sucrose.

It is also known that non-calorie sweeteners evoke different brain responses compared to calorie sugars. In particular, sucralose is known to display reduced ability to activate midbrain areas related to reward, including LHA, VTA, and NAc (164). Indeed, given the critical role of melanin-concentrating hormone (MCH) neurons in the LHA in establishing nutrient preference, a preclinical study showed that sucrose activated MCH neurons, resulting in dopamine release (DA). By contrast, sucralose was able to induce DA release in mice only in the presence of light stimulation, which led to the activation of MCH neurons. These findings suggest that non-calorie sweeteners require additional stimuli to obtain the same rewarding effect of sucrose (Figure 1) (165).

Significant controversy exists over the effects of low-calorie sweeteners on metabolic health. Studies conducted on rodents (166) and humans reported a positive association of low-calorie sweetener consumption with weight gain and/or diabetes (167–169), other studies a positive association with lower BMI and weight loss (170, 171), in other cases their use was not related to metabolic parameters (42, 43). Such heterogeneity is probably due to methodological limitations of some of these studies (172).

A recently published study shed some light on the controversy regarding the effects of low- and non-calorie sweeteners on metabolic health (129). Authors reported that consumption of sucralose was able to rapidly impair glucose metabolism and brain response to sweet taste in healthy subjects only when administered in the presence of carbohydrates. In fact, insulin sensitivity as well as neural responses to sugar were not altered by sucralose or carbohydrate alone. Notably, the combined effect of sucralose and carbohydrates was even more pronounced in adolescents, who showed a dramatic increase in insulin resistance measured by the Homeostatic Model Assessment for Insulin Resistance index (HOMA-IR). These findings refute the “sweet uncoupling hypothesis,” which is based on the concept that uncoupling sweet taste from caloric content could determine metabolic dysfunctions and reduce the potency of sweet taste (129).

These data may help explaining the obesogenic potential of low-calorie sweeteners in the context of western diets, especially considering the frequent use of “diet drinks,” often containing non-calorie sweeteners, associated with carbohydrates-rich meals.

## Effects of Sweeteners on Gut Hormones

**Figure 2:**
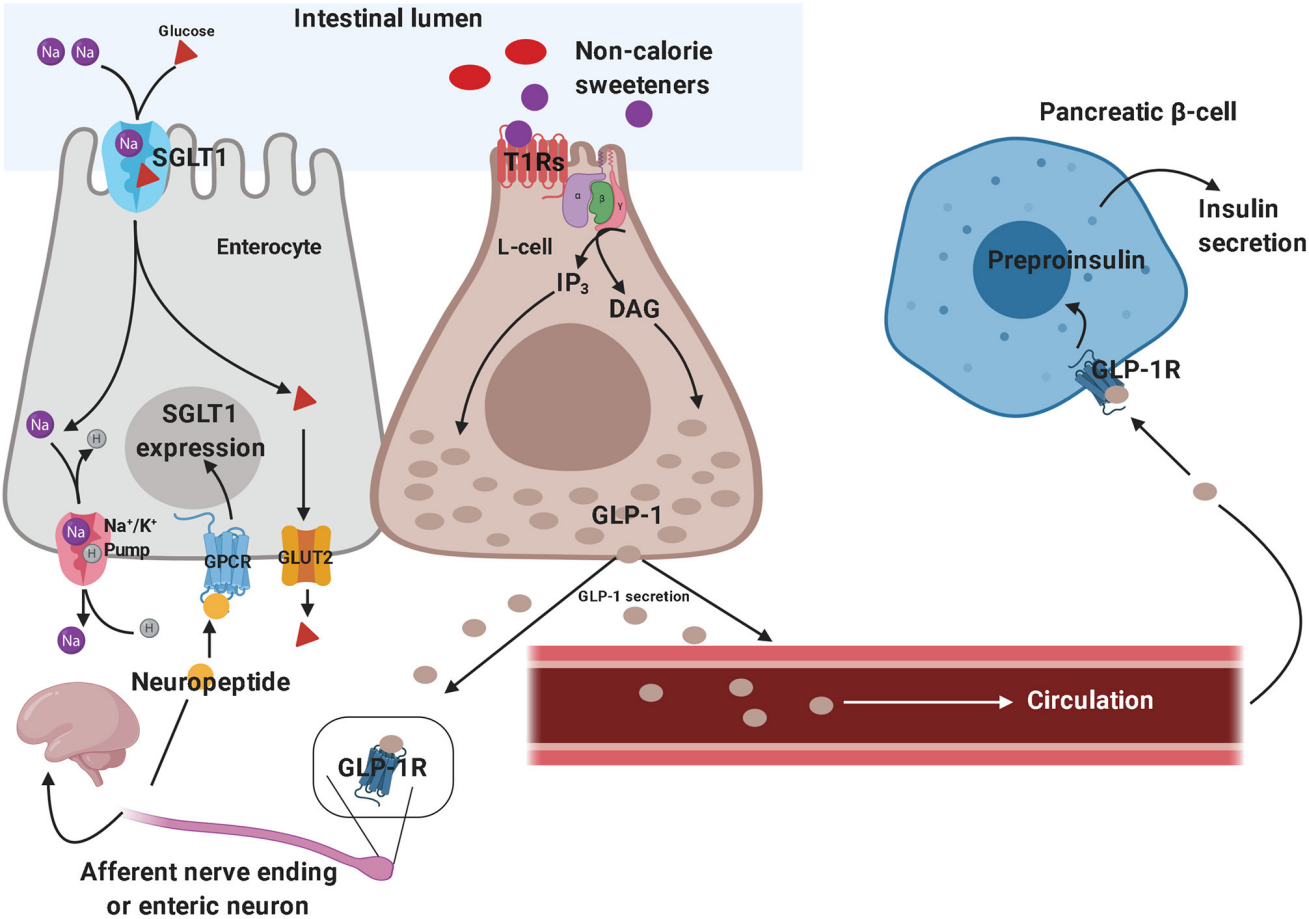
Effects of non-calorie sweeteners in the gastrointestinal tract. Non-caloric sweeteners bind to sweet-taste receptors (T1Rs) on enteroendocrine L-cells, promoting the synthesis of a series of second messengers, which ultimately results in GLP-1 release. GLP-1 stimulates the peripheral endings of afferent nerve fibers—which send GLP-1-signal toward the central nervous system—and promote neuropeptide release by enteric neurons, thus triggering the up-regulation of SGLT1 in enterocytes. Therefore, GLP-1 signaling ultimately results in increased intestinal glucose absorption. On the other hand, the role of circulating GLP-1 in eliciting glucose-dependent insulin secretion by pancreatic β-cells is well-established. These molecular mechanisms have been demonstrated both *in vitro* and *in vivo*, in preclinical studies, although they still need to be confirmed in clinical studies. GLP-1, glucagon-like peptide 1; SGLT1, sodium-dependent glucose cotransporter-1.

The sweet taste perception begins with the activation of taste receptors of the tongue, which are located within the taste buds of lingual papillae (173). Taste receptors include G protein-coupled receptors and ion channels. Type 1 taste receptors (T1Rs; sweet-taste and umami receptors) and type 2 taste receptors (T2Rs; bitter-taste receptors) are both G protein-coupled receptors (174, 175). The activation of these receptors generates second messengers, such as inositol trisphosphate and diacylglycerol, leading to the activation of taste-transduction channels (176). These metabolic pathways project to brain circuits, allowing the appreciation of taste. Sweet-taste receptors are also expressed outside of the oral cavity. They have been found throughout the gastrointestinal tract, particularly in the enteroendocrine L and K cells (177, 178). Besides the gastrointestinal tract, sweet-taste receptors have also been found in pancreatic β-cells (179), bile ducts (180), and lungs (181). In the gut, glucose is absorbed through sodium-dependent glucose cotransporter-1 (SGLT-1) localized on the luminal membrane and the passive glucose transporter 2 (GLUT2) on the basolateral membrane of the enterocyte (182). Glucose binding to sweet-taste receptors present on enteroendocrine cells leads to GLP-1 and peptide YY (PYY) secretion from L-cells, promoting satiety (183). Since both non-calorie and low-calorie sweeteners bind to sweet-taste receptors present in the oral cavity and subsequently lead to the sweet taste perception, it has been hypothesized that non-calorie sweeteners and low-calorie sweeteners may activate the same sweet-taste receptors expressed on enteroendocrine cells, promoting gut hormone secretion. *In vitro*, sucralose has been shown to stimulate GLP-1 secretion from a human L-cell line (NCI-H716 cells) (184) and to increase GLP-1 and glucose-dependent insulinotropic peptide (GIP) release in a murine enteroendocrine cell line (182) (Figure 2). However, these results were not confirmed *in vivo*. Several studies showed no effect of oral sucralose (185), aspartame, and acesulfame-K on GLP-1, PYY, ghrelin, or GIP secretion (186). These results have been confirmed also for sucralose administration before a solid meal, which did not elicit any effects on GIP or GLP-1 release (187). Nevertheless, other studies showed controversial results. In fact, non-calorie sweeteners ingested through diet soda have been shown to synergize with glucose to enhance GLP-1 release in healthy subjects, even if it is unclear whether this effect was due to the activation of sweet-taste receptors present on taste buds of lingual papillae and/or enteroendocrine cells, or if it was due to other mechanisms (188). Moreover, non-calorie sweeteners (such as sucralose, acesulfame-K and saccharin) have been shown to increase glucose absorption in the small intestine by up-regulating SGLT1 expression in mice through the activity of enteric neurons (182). Indeed, GLP-1 stimulates the release of neuropeptides, which in turn bind to G protein-coupled receptors (expressed on the basolateral membrane of enterocytes) and induce SGLT1 expression (182). *In vivo*, these sweeteners displayed the same effects by increasing GLUT2 expression at the level of the apical membrane (189). Giving the ability of non-calorie sweeteners to increase sugar absorption during a meal, it is worth considering their potential implications in terms of metabolic effects. The recent report that simultaneous consumption of sucralose and maltodextrin-derived glucose acutely disrupts glucose tolerance and insulin sensitivity (129) may be a likely consequence of the increased intestinal glucose absorption following the upregulated expression of SGLT1 and/or GLUT2.

*In vitro* studies demonstrated that sucralose, saccharin, and acesulfame-K stimulate pancreatic β-cell insulin secretion in the presence of glucose (179). The natural non-calorie sweetener stevia is also able to enhance glucose-induced insulin release, through inhibition of ATP-sensitive K-channels (190) and stevioside has been shown to reduce plasma glucose levels in mice (191), postprandial glucose and glucagon levels in subjects with type 2 diabetes (192). Finally, mechanistics studies are needed to better elucidate the effects of different sweeteners on gut hormone-mediated glucose homeostasis in humans.

Figure 2 depicts some of the effects of non-calorie sweeteners on enteroendocrine cells and gut hormones secretion, which have mainly been inferred from *in vitro* and animal studies.

## Impact of Sweeteners on Gut Microbiota

It has been hypothesized that the relationship between sugar, metabolic syndrome and its related disorders may be mediated, at least in part, by changes in the gut microbiota (193, 194). In particular, increased added sugar and novel sweeteners consumption may alter the carbohydrate pools in the gut, thus creating distinct environments that can favor adaptation and enhance colonization and virulence of some endogenous and/or exogenous pathogenic microbes (194).

Since most sugars and sweeteners are absorbed at the level of the small intestine by sugar transporters, only up to 30% of these compounds reach the large intestine (194). Therefore, the small intestine environment is more enriched in sweeteners compared to large intestine. Moreover, microbes in the small intestine have a greater number of carbohydrate uptake and utilization genes and transcripts compared to those in the large intestine (195).

Gut microbiota composition displays a remarkable spatial heterogeneity across the gastrointestinal tract, with different microbial communities creating distinct microenvironments at different gut locations (a phenomenon also known as “gut biogeography”) (196). Di Rienzi and Britton recently proposed that the variation in microbial communities found along the gastrointestinal tract can also depend on the variation in sugars and sweeteners present at different gut locations (194). Furthermore, changes in sugar and sweetener pool can lead to adaptation of the gut microbiota involving transcriptional, metabolic and compositional changes in gut microbes. These changes allow for increase in abundance of microbes whose niche and microenvironment are best filled. In addition, genetic changes can also allow existing microbes to alter their niche in order to better utilize the new nutrient pool (194). Suez et al. demonstrated that saccharin-fed mice developed glucose intolerance as a consequence of compositional and functional alterations to the gut microbiota (197). Similarly, germ-free mice receiving fecal transplantation from saccharin-fed animals displayed glucose intolerance, suggesting that derangements in glucose metabolism are mediated by saccharin-induced alterations to the gut microbiota (197). Similar findings were reproduced in healthy human subjects consuming saccharin for 1 week (197). Importantly, saccharin-fed mice showed a reduction in *Akkermansia muciniphila*, a mucin-degrading bacteria with probiotic properties associated with favorable metabolic effects (197). Saccharin consumption has also been shown to affect microbiota composition in rats; the growth of six bacterial strains (three *Lactobacillus* species and three *Escherichia coli* strains) was inhibited and the fermentation of glucose was decreased (198, 199). Mice treated with saccharin (0.3 mg/ml) for 6 months showed gut dysbiosis, which is broadly defined as any change in the composition of resident commensal microbial communities relative to the community found in healthy subjects (200), along with an increased expression of pro-inflammatory inducible NO synthase (iNOS) and TNF-α in liver (201).

Sousa et al. clearly demonstrated that a specific diet can alter gut microbiota by changing the abundance of specific strains. Of note, authors inoculated mice with an *Escherichia coli* strain carrying a mutation disrupting the ability to consume galactitol (a galactose-derived sugar alcohol) (202). Surprisingly, part of the *E. coli* population regained galactitol metabolism, resulting in the coexistence of two distinct strains within the gut microbiota that could or not consume galactitol (galactitol-positive and galactitol-negative strains, respectively) (202).

Apart from the role of sweeteners as nutrients for gut microbiota, it is worth considering that some of these compounds may exert direct toxict effects on specific microbes. For instance, xylitol cannot be metabolized by oral bacteria (203) and is therefore added to oral products or chewing-gums. Also, stevia glycosides (stevioside and rebaudioside A) have been shown to inhibit the growth of strains of *Lactobacillus reuteri*, a symbiotic *Lactobacillus* species which inhabits the gastrointestinal tract of mammals and is often administered as a probiotic additive in healthy foods (204). However, inulin and fructans—contained in the roots of stevia—favored the proliferation of *bifidobacteria* and *lactobacilli* in a pre-clinical study (205). Stevia also showed a bactericidal effect on enterohemorrhagic *Escherichia coli* (206). Also, some sugar alcohols have been shown to promote an increase in the number of beneficial gut microbes both in rats and in healthy human volunteers (207, 208). Mice fed a high-fat diet plus xylitol showed a reduced fecal content of *Bacteroidetes* and *Barnesiella*, along with an increased abundance of *Firmicutes* and *Prevotella* (209). Moreover, xylitol promoted a substantial change in rodent fecal microbiota, decreasing gram-negative and increasing gram-positive bacteria (210).

Of note, the ability of sweeteners to affect gut microbiota composition may also have important immunological implications. In this regard, a recent study conducted on streptozotocin-induced diabetic mice and non-obese diabetic (NOD) mice—which are both widely used as animal models of human type 1 diabetes—has shown that trehalose, a natural caloric disaccharide derived from a rodent intestinal nematode and characterized by antioxidant properties (211), is able to affect gut microbiota by increasing the abundance of *Ruminococcus* spp. (212). Such change in intestinal microbiota composition appears to account for the induction of CD8+ regulatory T cells, which play a role in inhibiting the onset of diabetes and reducing blood glucose levels in diabetic animals (212). In addition, authors found that patients with type 1 diabetes, when compared to healthy volunteers, had fewer CD8+ regulatory T cells, as well as lower serum trehalose concentrations and fecal content of *Ruminococcus* (212). These results suggest that trehalose may have a potential prophylactic and/or therapeutic role in humans (e.g., use of trehalose and *Ruminococcus* strains as a prebiotic and probiotic, respectively), as a tool to induce CD8+ regulatory T cells in order to prevent the development of type 1 diabetes and/or counteract the immune-mediated β-cell destruction shortly after the onset of the disease.

Since sweeteners cross the placenta (213) and are found in the maternal milk (214), there is the potential for a relationship between prenatal exposure to different sweeteners and gut microbiota composition later in life. Animal studies indicate that acesulfame-K crosses the placenta during pregnancy and can potentially lead to an increased sweet preference during adulthood (215). On the other hand, prenatal sucralose exposure does not affect fetal organogenesis (216), but increases the risk for hematopoietic neoplasia in male mice (135) and favors adipocyte differentation in cultured pre-adipocytes (217). These results suggested that non-calorie sweeteners consumption during pregnancy could impact on offspring adipose tissue differentiation, promoting childhood obesity (217). Moreover, exposure of pregnant mice to a mixture of acesulfame-K and sucralose at different concentrations demonstrated that non-calorie sweeteners were able to affect gut microbiota composition in the offspring in a dose-dependent manner, increasing *Firmicutes* content and reducing the amount of beneficial species, including *Akkermansia muciniphila* (218). In conclusion, early prenatal exposure to specific non-calorie sweeteners could favor the occurrence of metabolic diseases later in life, by inducing detrimental changes in the gut microbiota composition.

The aforementioned findings support the notion that sweetener consumption modifies the nutrient environment in the gut and induces a series of functional changes in the gut microbiota, which potentially result in transcriptional, metabolic, compositional, and/or genetic adaptation by gut microbes. In turn, microbial adaptation to sweeteners may affect host-microbe interaction and influence the subsequent immune responses (e.g., pro-inflammatory, anti-inflammatory, immune responses that promote microbe survival or clearance) (194). However, the mechanisms underlying the microbial adaptation to a given sweetener are not fully understood yet. In particular, it is still not clear if dietary sugars and sweeteners can also induce changes in the host environment. The exact impact on the host exerted by microbial metabolites derived from added sugar and sweetener metabolism also needs to be clarified and addressed in future studies.

## Health Concerns Related to The Use of Sweeteners

To date, the European Union (EU) and EFSA approved the use of 11 non-calorie sweeteners, namely: acesulfame-K (E-950), advantame (E-969), aspartame (E-951), aspartame-acesulfame salt (E-962), cyclamic acid and its sodium and calcium salts (E-952), neohesperidin dihydrochalcone (E-959), neotame (E-961), saccharin (E-954), stevia (E-960), sucralose (E-955), and thaumatin (E-957). EU and EFSA confirmed that non-nutritive and low-calorie sweeteners are safe for human health if used within the ADI (219).

Amongst low-calorie sweeteners, EU approved the following compounds: sorbitol and sorbitol syrup (E420), mannitol (E-421), isomaltose (E-953), polyglycitol syrup (E-964), maltitol and maltitol syrup (E-965), lactitol (E-966), xylitol (E-967), and erythritol (E-968).

Unlike non-caloric sweeteners, polyols and low-calorie sweeteners are classified as GRAS and ADI is not reported for them (219). An international consensus statement on the use of low- and non-calorie sweeteners has been signed in Lisbon in July 2017 (220). The Consensus concluded that low- and non-calorie sweeteners consumption is safe, as also supported by WHO, FDA (221) and EFSA (222), and the dietary consumption of low- and non-calorie sweeteners promotes dental health when these compounds replace free sugars (223, 224). Therefore, the Consensus encourages the education of consumers on the use of products containing low- and non-calorie sweeteners, in order to increase awareness of general population on their correct use (225).

Finally, consumption of low- and non-calorie sweeteners during pregnancy showed neutral effects on offspring health and data obtained from animal studies were not confirmed in humans (226).

Although more research is needed to fully assess the effects of *in utero* exposure to sweeteners, current evidence does not suggest adverse effects in pregnancy. Nevertheless, it is recommended that sweeteners are consumed in moderate amounts, adhering to the acceptable daily intake standards established by regulatory agencies (213).

## Discussion

FDA has approved several types of sugar substitutes, considering them as safe. Nonetheless, the *American Heart Association* and the *American Diabetes Association* suggest to limit the use of sweeteners due to the lack of strong evidence for their effects on body weight and cardiometabolic risk factors in the long-term period (227).

With regard to effects on gut microbiota, most of the sweeteners affect bacterial gut composition, potentially inducing dysbiosis. Among sweeteners, polyols seem to show a good safety profile. Moreover, they are non-cariogenic, do not negatively affect gut microbiota and are characterized by a very low-energy value (45). Moreover, the potential favorable effects of polyols on glucose homeostasis may suggest their use as a valid option in subjects with type 2 diabetes and metabolic syndrome, although further research is needed in this area.

In this context, a careful nutritional advice is essential for a conscious use and for a correct transition, through the use of sweeteners, from sweetened foods to sugar-free foods. The role of nutrition specialists appears therefore crucial to recommend a diet with a proper use of sweeteners, avoiding the risk of an excessive use of these compounds.

Given the scarcity of data on sweetener safety in the long-term period, it is important to carefully evaluate the use of these compounds particularly in selected patients, such as those affected by metabolic derangements. Indeed, different studies and meta-analyses found an association between the consumption of sweeteners and artificially sweetened beverages with increased risk of overweight, obesity, metabolic syndrome and type 2 diabetes (86, 163, 228–230), thus highlighting the need for future prospective studies aimed at evaluating the exact impact of different types of sweeteners on human health from a metabolic perspective. However, when consumed in moderate amounts, sweeteners may be used as part of a nutritional rehabilitation program aimed to limit daily consumption of refined sugars (86, 163, 228–230).

Over the last decades several healthy dietary patterns have been proposed to tackle the growing obesity epidemic. Dietary approaches based on marked reduction of carbohydrate and refined sugar consumption are emerging in clinical practice and are highly debated. During the last few years, interest in very low-calorie ketogenic diets (VLCKD) has gradually grown due to their safety and their marked potential in inducing weight loss (231). In this context, non-calorie sweeteners represent a valuable tool for improvement of patient adherence to a strict nutritional regimen and rehabilitation program. The addition of non-calorie sweeteners to food replacements allowed for a marked reduction in carbohydrate and sugar content (<30 g/day, ≃13% of total energy intake), with preservation of food palatability and diet satisfaction (231), thereby avoiding craving and increase in appetite, which may reduce the efficacy of a VLCKD. On the basis of the recent findings on the effects of sucralose (129), the association of non-calorie sweeteners to a very low-carbohydrate nutritional regimen represents a valid approach to prevent the detrimental metabolic effects on insulin sensitivity and the altered neural response to sugars induced by an excessive carbohydrate consumption (Figure 1).

## Concluding Remarks

Dietary consumption of sweeteners has progressively increased over the last decades in order to reduce the burden of cardiovascular and metabolic diseases caused by modern western diets, which are characterized by a high content in refined and added sugars. Also, the introduction of sugar taxes in several countries is likely to cause an even greater use of these compounds.

Nonetheless, at present there is still scarce evidence to establish conclusively whether the consumption of different types of sweeteners (e.g., low-calorie sweeteners vs. non-calorie sweeteners) can result in significant beneficial or detrimental effects on energy balance, appetite, body weight, and/or cardiometabolic risk factors in healthy subjects and patients with metabolic diseases (particularly obesity and type 2 diabetes). Indeed, the health impact of sweetener consumption, as well as the potential health consequences resulting from switching from one sweetener to another, still remain poorly understood. Therefore, future prospective studies aimed to address short- and long-term safety and efficacy of different types of sweeteners in various clinical settings (e.g., obesity, type 2 diabetes, metabolic syndrome) and in different age groups are needed.

Another area that warrants further investigation is the impact of different types of sweeteners on gut microbiota. Emerging evidence supports how different food components (including sweeteners) can drive changes in the gut microbiota, resulting in relevant implications for human health and disease (86, 194, 230, 232, 233). Mechanistic studies using gut organoids or animal models will certainly help to better elucidate: (i) how different types of sweeteners can reshape the gut microbiota, (ii) the interactions between sweeteners/sweetener metabolites, gut microbiota and host, and (iii) the consequences of these interactions on host physiology and biological processes in the short- and long-term period. Future studies will also be helpful to evaluate which sweeteners are able to promote the growth of beneficial or detrimental gut microbes, resulting in potential human health benefits or harms. Additionally, clinical studies evaluating the impact of different sweeteners on gut microbiota composition will further help to fully address all these unanswered questions regarding the sweetener-gut microbiota-host triad.

Finally, the extent to which all the aforementioned sweetener-induced changes at different levels (central nervous system circuits, gut hormone secretion and gut microbiota) are clinically relevant in terms of human health is still not clear. Genetic, anthropometric and dietary differences may, at least in part, account for the high interindividual variability in the response to different types of sweeteners (233). Future studies based on epidemiological approaches combined with tools used in precision medicine may help to better establish the subset of individuals who are more likely to receive benefit or harm from sweetener consumption (233).

## Author Contributions

All authors listed have made a substantial, direct and intellectual contribution to the work, and approved it for publication. MC and EM conceived, wrote, and revised the manuscript. AFe and VM prepared the figures and wrote part of the manuscript. MI wrote part of the manuscript and carefully revised it. ML and AFa revised the manuscript.

## Conflict of Interest

The authors declare that the research was conducted in the absence of any commercial or financial relationships that could be construed as a potential conflict of interest.
